# Reversal of senescence-associated beta-galactosidase expression during in vitro three-dimensional tissue-engineering of human chondrocytes in a polymer scaffold

**DOI:** 10.1038/s41598-021-93607-9

**Published:** 2021-07-07

**Authors:** Shojiro Katoh, Atsuki Fujimaru, Masaru Iwasaki, Hiroshi Yoshioka, Rajappa Senthilkumar, Senthilkumar Preethy, Samuel J. K. Abraham

**Affiliations:** 1 Edogawa Evolutionary Lab of Science, Edogawa Hospital Campus, 2-24-18, Higashi Koiwa, Edogawa-Ku, Tokyo, 133-0052 Japan; 2 Department of Orthopaedic Surgery, Edogawa Hospital, 2-24-18, Higashi Koiwa, Edogawa-Ku, Tokyo, 133-0052 Japan; 3 Centre for Advancing Clinical Research (CACR), University of Yamanashi-Faculty of Medicine, 1110, Shimokato, Chuo, Yamanashi 409-3898 Japan; 4 Mebiol Inc., 1-25-8, Nakahara, Hiratsuka, Kanagawa 254-0075 Japan; 5 The Fujio-Eiji Academic Terrain (FEAT), Nichi-In Centre for Regenerative Medicine (NCRM), PB 1262, Chennai, Tamil Nadu 600034 India; 6 The Mary-Yoshio Translational Hexagon (MYTH), Nichi-In Centre for Regenerative Medicine (NCRM), PB 1262, Chennai, Tamil Nadu 600034 India; 7 JBM Inc., 3-1-14, Higashi Koiwa, Edogawa-Ku, Tokyo, 133-0052 Japan; 8 Antony- Xavier Interdisciplinary Scholastics (AXIS), GN Corporation Co. Ltd., 3-8, Wakamatsu, Kofu, Yamanashi 400-0866 Japan

**Keywords:** Regenerative medicine, Tissue engineering, Senescence

## Abstract

Regenerative medicine applications require cells that are not inflicted with senescence after in vitro culture for an optimal in vivo outcome. Methods to overcome replicative senescence include genomic modifications which have their own disadvantages. We have evaluated a three-dimensional (3D) thermo-reversible gelation polymer (TGP) matrix environment for its capabilities to reverse cellular senescence. The expression of senescence-associated beta-galactosidase (SA-βgal) by human chondrocytes from osteoarthritis-affected cartilage tissue, grown in a conventional two-dimensional (2D) monolayer culture versus in 3D-TGP were compared. In 2D, the cells de-differentiated into fibroblasts, expressed higher SA-βgal and started degenerating at 25 days. SA-βgal levels decreased when the chondrocytes were transferred from the 2D to the 3D-TGP culture, with cells exhibiting a tissue-like growth until 42–45 days. Other senescence associated markers such as p16^INK4a^ and p21 were also expressed only in 2D cultured cells but not in 3D-TGP tissue engineered cartilage. This is a first-of-its-kind report of a chemically synthesized and reproducible in vitro environment yielding an advantageous reversal of aging of human chondrocytes without any genomic modifications. The method is worth consideration as an optimal method for growing cells for regenerative medicine applications.

## Introduction

Cell and tissue engineering-based regenerative therapies warrant good-quality cells and tissues for optimal clinical outcomes. Cellular senescence is a multifaceted process that arrests cell proliferation^1^. The term was first mentioned in the landmark paper by Leonard Hayflick, who reported that in vitro cultured primary human fibroblasts have a restricted lifespan, which is approximately 50 cell divisions, known as “Hayflick’s limit”^2^. A tissue’s ability to regenerate decreases when a significant proportion of proliferating cells in it undergoes cellular senescence. The number of senescent cells increases with age in multiple types of tissues^3^. Cellular senescence is triggered in response to a variety of stressors, including telomere shortening, oxidative stress, DNA damage and oncogene activation^4^. Telomere shortening is the major cause underlying replicative senescence^5^. While most human somatic cell types express little or no telomerase activity, leading to telomere loss, and proliferating normal stem cells though express regulated telomerase, the expression level is insufficient to maintain telomeres, and gradual telomere erosion occurs. Progressive telomere shortening leads to in vitro replicative senescence^6^. Regarding age-related diseases like osteoarthritis (OA), chondrocytes primarily are thought to play a major role in OA induction as they become senescent due to progressive telomere shortening with age. Senescent chondrocytes are absent from normal cartilage and are present near osteoarthritic lesions^7^. When such senescent cells were transplanted into the knee joint of wild type mice, an OA-like state was induced, thus showing that senescence of chondrocytes is a major factor driving OA^8^. When chondrocytes are cultured in vitro, especially in monolayer, they easily lose their native phenotype, de-differentiate and express various senescence- and dedifferentiation-related genes. Replicative senescence in vitro has been observed after 30–40 passages during in vitro culture of chondrocytes, which then exhibit features of the senescent phenotype, including enlarged flattened cells in culture and the expression of SA-βgal^9^. The cellular senescence of in vitro cultured cells is usually overcome by inducing telomerase activity or initiating recombination-mediated alternative lengthening of telomeres (ALT) pathway(s) or genomic modifications such as reprogramming using specific transcription factors, all of which carry a risk of oncogenesis^10^. An in vitro culture method which does not involve any such genomic modifications would be ideal for use in regenerative therapies. The capabilities of a three-dimensional (3D) thermo-reversible gelation polymer (TGP) to maintain the native phenotype for a longer time in vitro have been reported for several cell types such as corneal endothelial precursor cells^11^, corneal limbal stem cells^12^, mesenchymal stem cells^13^, buccal epithelial cells^14^ and chondrocytes^15–18^. This 3D-TGP can maintain the native hyaline phenotype of knee-cartilage-derived chondrocytes from bovine^15^, rabbit^16^ and human^17,18^ sources for 16–18 weeks, both in vitro and in vivo. In vitro 3D-TGP-tissue-engineered cartilage tissue expressed pluripotency-related markers in a lectin micro-array^18^, higher miRNA21 and 140 expression indicative of healthy cartilage phenotype^19^ and mesenchymal-chondroprogenitor markers^20^. We sought to examine the expression of senescence-associated beta-galactosidase (SA-βgal) in human chondrocytes derived from elderly donors affected by OA, cultured in 2D- followed by 3D-TGP.

## Methods

The institutional ethics committee of Edogawa Hospital, Tokyo, Japan, approved the study. Discarded cartilage biopsy tissues were obtained from elderly patients with severe OA (aged between 60 and 85 years) who underwent arthroscopy and were employed in the study. The study was conducted in accordance with relevant guidelines/regulations, and informed consent was obtained from all of the participants and/or their legal guardians.

The cartilage tissue samples were subjected to chondrocyte isolation and culture, following our methodology reported earlier^16–18^. Nine samples were used in the study. The cartilage tissues were subjected to digestion with 0.25% trypsin for 30 min in an orbital shaker at 150 rpm, at 37 °C, followed by digestion in 2 mg/ml collagenase digestion for 12–18 h at 37 °C in an orbital shaker. After the digestion, the cells were isolated by filtering with a 100-μm cell strainer. The cells were centrifuged at 1000 rpm for 10 min and cultured in two-dimensional (2D) monolayer using media containing low-glucose DMEM, 10% autologous plasma, 1% penicillin streptomycin, 50 μg/ml gentamicin and 0.25 μg/ml amphotericin B and L-ascorbic acid (50 mg/ml) for 17–25 days at 37 °C with 5% CO_2_. After 17–25 days when the 2D-cultured cells started degenerating, a portion of the cells was seeded into the 3D-TGP scaffold in a cylindrical silastic tube for culture using high-glucose DMEM, 10% autologous plasma, 1% penicillin streptomycin, 50 μg/ml gentamicin and 0.25 μg/ml amphotericin B and L-ascorbic acid (50 mg/ml), placed on an orbital shaker at 80 RPM in 5% CO_2_ incubator, while the other portion was continued as 2D culture. The 3D cultures were maintained for 42–45 days. For the tissue harvest from 3D-TGP, the culture supernatant was removed and cold phosphate buffered saline (PBS) kept at 4° C was added to liquify the TGP and the tissue like construct was retrieved.

For histological staining, the 2D and the 3D-TGP cultures were fixed using formalin, and then embedded in paraffin blocks. Serial sections were deparaffinized and stained with haematoxylin and eosin (H&E), safranin O/fast Green and toluidine blue employing standard histological techniques. The 2D-cultured cells were subjected to SA-βgal measurement before transfer to 3D-TGP and on days 22–45, in whichever sample they did not degenerate while the 3D-TGP- cultured cells were subjected to SA-βgal measurement on days 26, 36 and 42–45 of culture. The 2D and 3D-TGP cultures were also subjected to evaluation for p16^INK4a^ and p21 mRNA expression.

**Figure 1:**
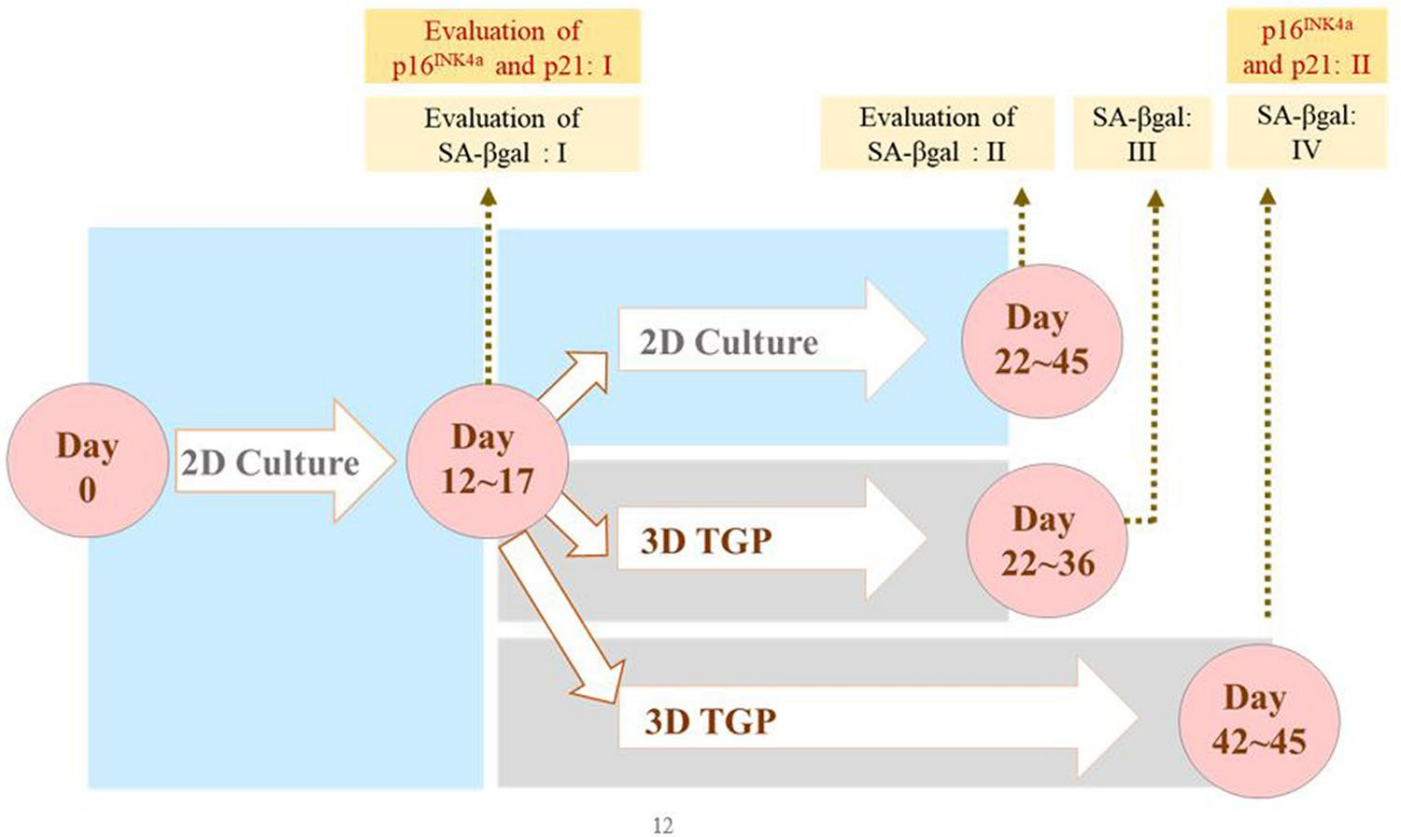
Illustration of the study groups and time-points of evaluation of SA-βgal, p16INK4a and p21 in two-dimensional (2D) and three-dimensional (3D) thermo-reversible gelation polymer (3D-TGP) cultures.

The time-points of measurement in 2D and 3D-TGP are illustrated in Fig. 1.

For SA-βgal measurement, cells from the 2D and 3D-TGP cultures were stained using a SPiDER-βgal cellular senescence plate assay kit (Dojindo Laboratories, Japan) to measure their SA-βgal. For the 3D-TGP cultured cells, single cells were obtained from spheroids harvested from the 3D culture by incubation with Collagenase Type II (1 mg/ml) for 2 min. The cell culture supernatant from the cells in the cell culture plate/dish was removed, and the cells were washed with phosphate buffered saline (PBS) once. Lysis buffer was added, and the plate/dish was incubated at room temperature for 10 min. The lysate solution was added to each well of the cell culture plate. Then, the SPiDER-βgal working solution was added to each well and incubated at 37 °C for 30 min. The stained cells were measured with FACSVia and analysed with the FlowJo software (BD).

The cells from 2 and 3D-TGP were also subjected to qRT-PCR for the expression of other senescence associated markers p16 ^INK4a^ and p21^21,22^.

Total RNA was isolated and RT-PCR was performed using the One Step TB Green PrimeScript PLUS RT-PCR Kit (Perfect Real Time; Takara, Japan) and Thermal Cycler Dice Real Time System Lite (TP700, TaKaRa).

The primers employed are provided below.

p16 ^INK4a^

5′ to 3′: GGCACCAGAGGCAGTAACCA

3′ to 5′: CCTACGCATGCCTGCTTCTACA

p21

5′ to 3′: GCGATGGAACTTCGACTTTGT

3′ to 5′: GGGCTTCCTCTTGGAGAAGAT

For mRNA quantification, the relative expression of the genes of interest was normalized against the GAPDH housekeeping gene by employing the comparative cycle threshold (Ct) method.

∆Ct = Ct (gene of interest) – Ct (housekeeping gene (GAPDH))

∆∆Ct = ∆Ct (treated sample or experimental sample) – ∆Ct (control sample)

All of the data were analysed using the Microsoft Office Excel software package. Student’s paired t-tests were also calculated using this package. P-values < 0.05 were considered significant.

### Ethical approval

The institutional ethics committee of Edogawa Hospital, Tokyo, Japan approved the study.

## Results

**Figure 2:**
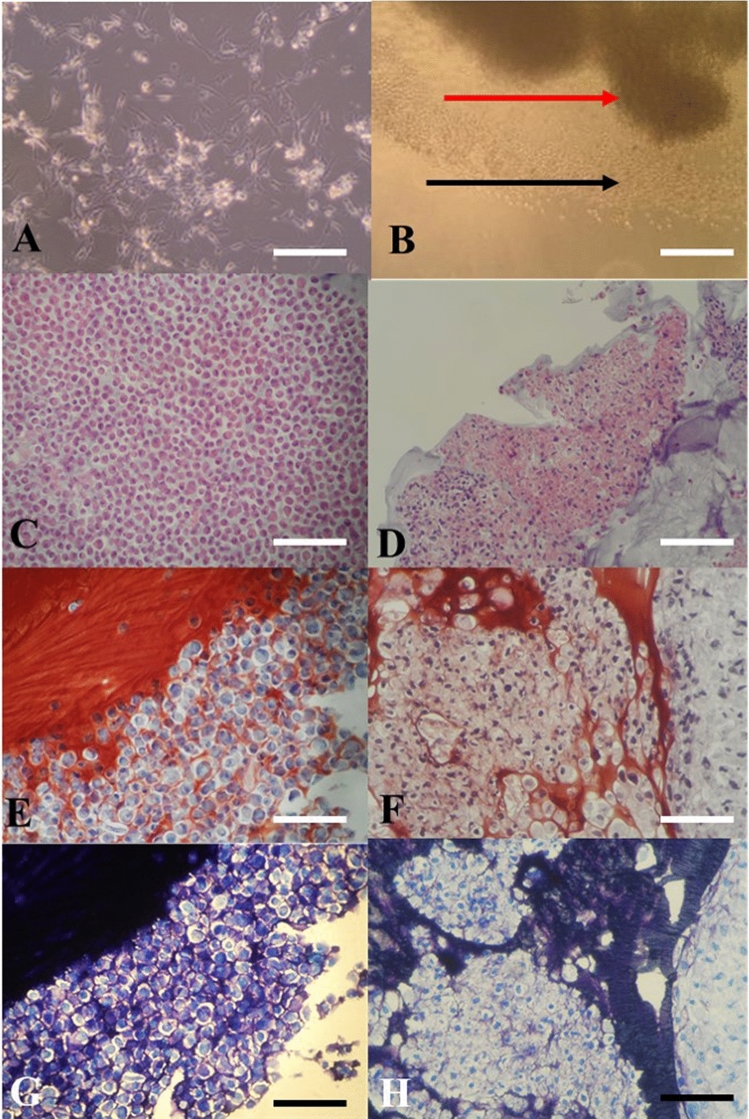
(**A**,**B**) In vitro culture images: (**A**) Chondrocytes in two-dimensional (2D) culture de-differentiating into fibroblast-like cells; (**B**) In vitro cultured chondrocytes growing in a tissue-like manner in three-dimensional (3D) thermo-reversible gelation polymer (3D-TGP) culture (the red arrow indicates the tissue; the black arrow indicates the cells migrating out into the 3D environment into the tissue); (**C**,**D**) H-and-E staining images: (**C**) Chondrocytes in 2D observed as individual cells. (**D**) 3D-TGP tissue-engineered chondrocytes exhibiting continuous tissue morphology with hyaline phenotype; (**E**,**F**) Safranin O/Fast Green staining images: (**E**) Chondrocytes in 2D observed as individual cells. (**F**) 3D-TGP tissue-engineered chondrocytes exhibiting continuous tissue morphology; (**G**,**H**) Toluidine blue images: (**E**) Chondrocytes in 2D observed as individual cells. (**F**) 3D-TGP tissue-engineered chondrocytes exhibiting continuous tissue morphology (All scale bars = 100 μm).

The cells grew individually in a monolayer 2D culture with de-differentiating into fibroblast like cells and could be maintained in the in vitro culture in a healthy manner for only up to 25 days after which they started showing signs of degeneration. After transfer to 3D-TGP, the cells in the 3D-TGP grew as a tissue-like morphology with native hyaline phenotype maintenance observed in H&E staining, Safranin O/Fast Green and Toluidine blue throughout the culture period of 42–45 days (Fig. 2).

**Figure 3:**
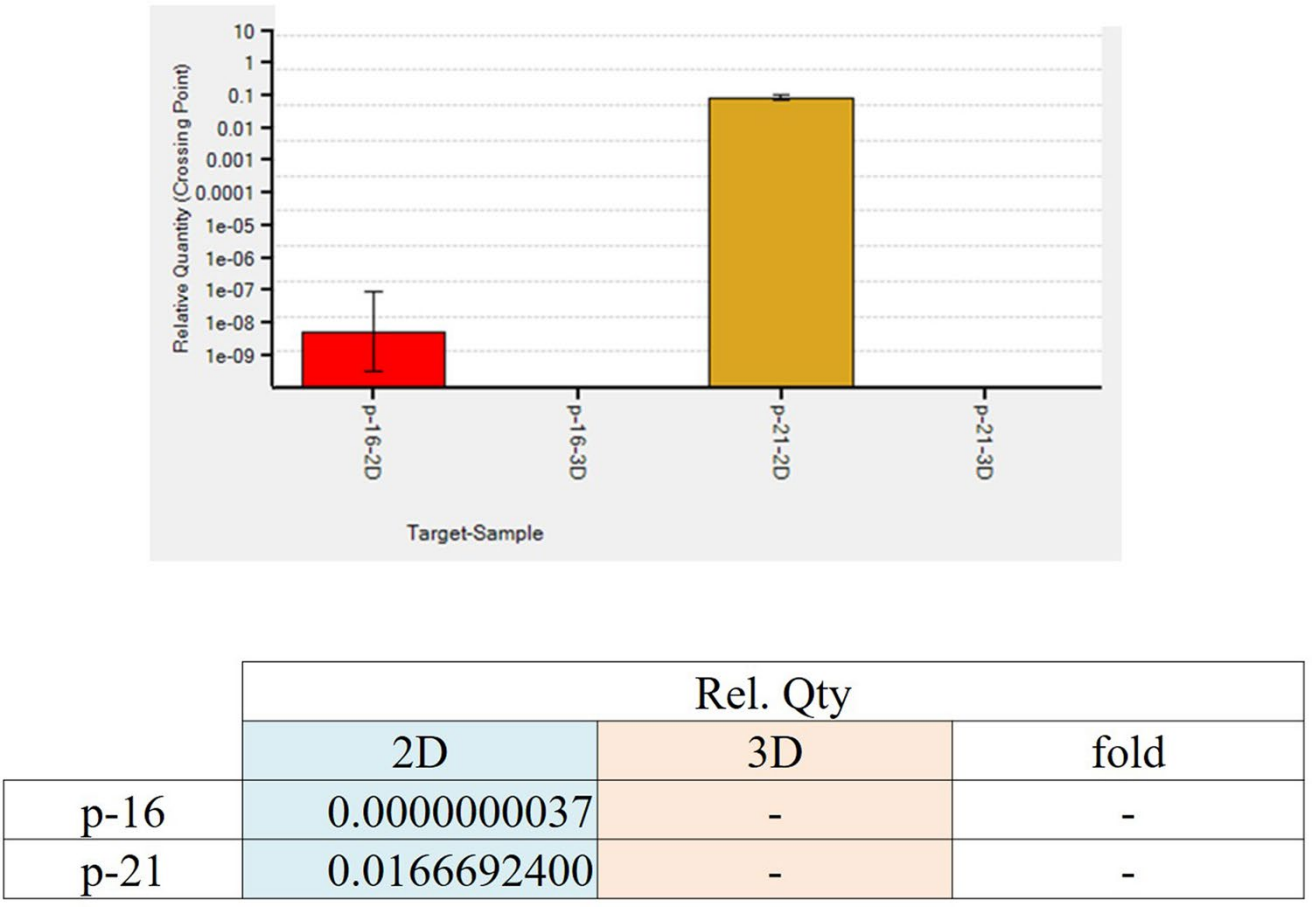
Relative expression of p16 ^INK4a^ and p21 only in 2D cultured chondrocytes and not in 3D-TGP indicating presence of senescent cells in 2D cultures but not in 3D-TGP.

**Figure 4:**
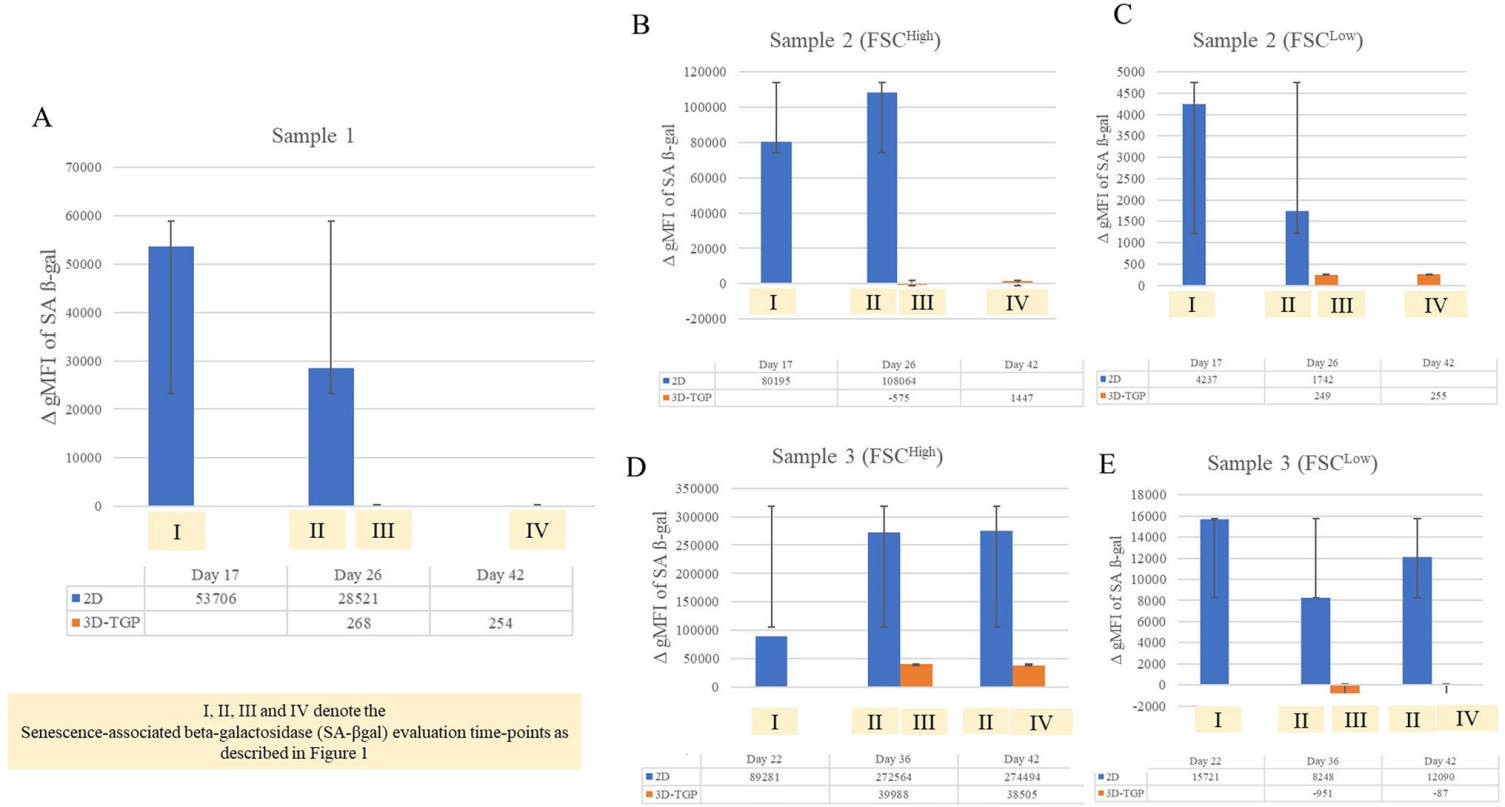
Mean fluorescence intensity (ΔG MFI) of the expression of SA-βgal evaluated by flow cytometry in 2D compared to 3D-TGP at different durations of culture with 2D-cultured chondrocytes (evaluation I and II) showing higher levels of SA-βgal as culture, while the 3D-cultured cells (evaluation III and IV) showed very low levels of SA-βgal throughout the culture period. (**A**) Sample 1; (**B**) Sample 2 (FSC^(High)^); (**C**) Sample 2 (FSC^(Low)^); (**D**) Sample 3 (FSC^(High)^); (**E**) Sample 3 (FSC^(Low)^). I, II, III and IV denote the Senescence-associated beta-galactosidase (SA-βgal) evaluation time-points as described in Fig. 1.

**Figure 5:**
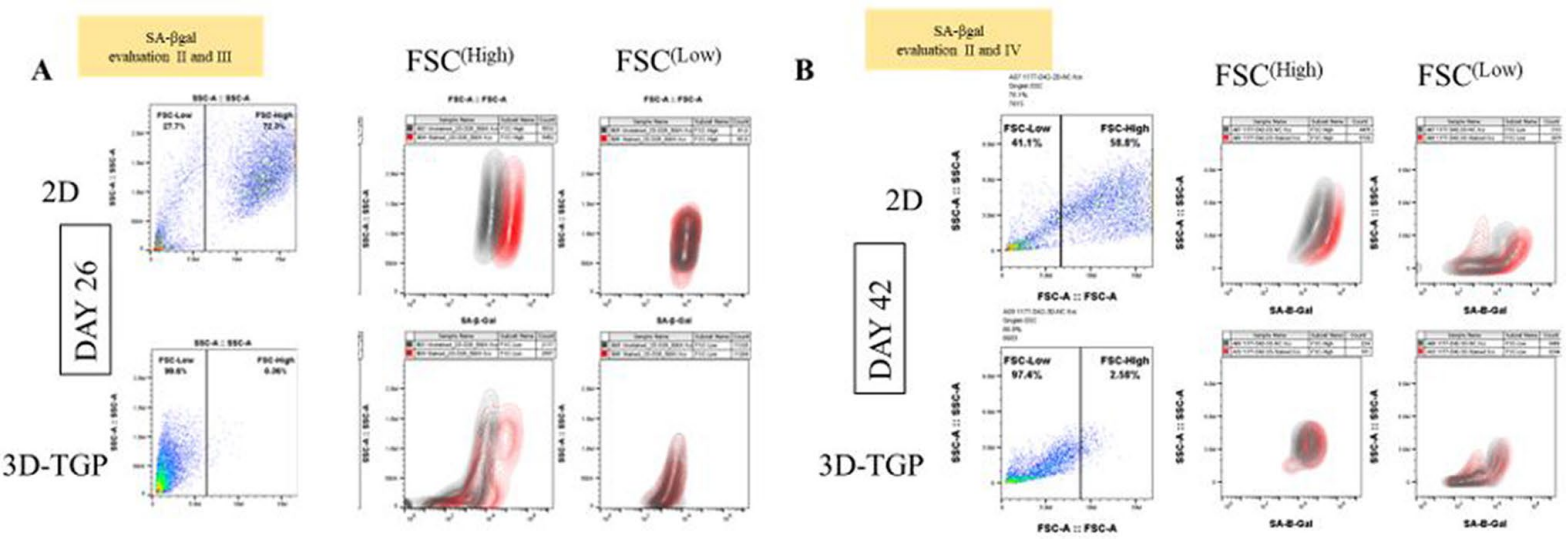
Gating in the flow cytometric analysis of osteoarthritic chondrocytes grown in 2D and 3D-TGP with the SA-βgal expression in two heterogeneous populations sorted by flow cytometry (FSC^high^ versus FSC^low^) with the 2D-grown cells showing higher SA-βgal expression than 3D-TGP cultured cells on both day 26 and day 42 of culture (**A**) Day 26; (**B**) Day 42. II, III and IV denote the Senescence-associated beta-galactosidase (SA-βgal) evaluation time-points as described in Fig. 1.

Regarding the mRNA expression of p16 ^INK4a^ and p21, they were expressed only in 2D cultured samples and not in 3D-TGP indicating presence of senescent cells in 2D cultures but not in 3D-TGP (Fig. 3). The average delta G of mean fluorescence intensity (ΔG MFI) of the expression of SA-βgal in the cells from 2D culture was 42,016.6 while after transfer to 3D-TGP the value greatly decreased to an average of − 144.66 (Fig. 4) and it only slightly increased after 42–45 days of culture in 3D-TGP. These values are of the larger cells observed during gating in the flow-cytometry (the forward scatter [FSC]^high^ group) (Fig. 5). For another population (FSC^low^ group), the average of ΔG MFI was 190,314 on day 17 in 2D, which decreased to 19,706.5 after transfer to 3D-TGP and again only slightly increased till day 42–45 in 3D-TGP (Figs. 4, 5). The difference between the higher expression of total ΔG MFI of SA-βgal in cells from 2D compared to decreased expression in 3D-TGP was however not statistically significant (p-value = 0.089723).

## Discussion

The most widely used biomarker for identifying senescent and aging cells in vitro is SA‐β‐gal, which is the activity of an enzyme called β‐galactosidase, which is detectable at pH 6.0 in cells undergoing replicative or induced senescence in vitro. It is absent in proliferating cells^23^. SA-βgal originates from lysosomal β‐galactosidase activity, which increases in senescent cells due to increased lysosome content, whose levels become detectable at pH 6.0 when it surpasses a threshold limit^23^. Relevant to chondrocytes and aging, a study which compared the SA-βgal expression of normal (controls) versus OA cartilage reported that no SA-βgal staining was observed in the normal articular cartilage samples, while the percentage of SA-βgal-positive chondrocytes was 13.00 ± 5.77% in mild lesions, 31.65 ± 6.91% in moderate lesions and 51.95 ± 6.21% in severe lesions^24^, thus implying that SA-β-gal expression is associated with progressive knee joint damage from OA and is a potential indicator of disease severity^24^. Another study also reported that cultured chondrocytes isolated from near the lesion sites of OA contained a greater percentage of SA-β-gal-positive cells than cultures isolated from distal sites or normal cartilage did^25^. In the present study, the 2D monolayer culture led to rapid replicative senescence in 17 days, evident from the higher levels of SA‐β‐gal expression (Fig. 4), expression of p16 ^INK4a^ and p21 (Fig. 3) compared to 3D-TGP, whose environment re-differentiates the de-differentiated fibroblasts to yield younger cells that grow as a tissue maintaining the native hyaline phenotype (Fig. 2) for a longer period. Having shown earlier that chondrocytes grown in TGP express pluripotency-associated markers in a lectin microarray, the current study further substantiates the presence of a heterogeneous cell population (Fig. 5) containing FSC^high^ and FSC^low^, with FSC^low^ comprising smaller cells, presumably progenitor cells, which needs validation, apart from the study of other markers of senescent cells such as γ-H2AX, the formation of senescence-associated heterochromatin foci (SAHF) and the acquisition of a senescence-associated secretory phenotype (SASP)^26^. Both in vitro and in vivo studies have reported that senolytics which eliminate senescent cells improve physiological function in tissues and decrease aging^27^. These drugs comprise natural products, synthetic small molecules and peptides that target proteins involved in senescent-cell anti-apoptotic pathways (SCAPs). However, they are not free of side effects^23^, and any interference with the natural mechanisms lead to risks of genomic mutations and tumorigenicity. Here, we have employed a synthetic polymer which could help to grow younger cells in vitro for more efficient tissue-engineering and regenerative medicine applications. TGP maintains the native phenotype of cells without inducing gene abnormalities^13^, and its safety has been established in pilot clinical studies on humans^13,14,28^. A recent study^28^ has reported that there is age-associated cell-intrinsic defects in hematopoietic stem cells (HSCs) which cannot be restored by rejuvenating the niche alone. In the present study, the cells’ SA-βgal expression could be reversed by using a 3D-Poylmer scaffold which probably restores both the niche and the age-related intrinsic changes in the cells; however this needs further validation. The present study is only a preliminary study on the possibility of employing 3D-TGP culture technology for the in vitro reversal of aging by using a 3D polymer without using genomic modifications/proteins which may have other unwanted adverse effects. The technology’s efficacy can be confirmed further by studying the telomere length of the cells cultured in 2D- and 3D-TGP, in studies that are underway by our team.

## Conclusion

This is the first study to provide proof-of-concept evidence that in vitro cellular senescence analysed by SA-βgal expression can be reversed through culturing in a 3D-TGP scaffold-based culture platform that provides an environment which nurtures cells in the native phenotype, apart from making them younger by decreasing the senescence-associated characteristics, importantly without use of genomic modification techniques. This study opens a new avenue for evaluating techniques such as 3D-TGP to reverse in vitro aging, in order to produce younger good-quality cells for regenerative medicine applications.
